# Integrating community pharmacists in tuberculosis infection care: challenges and strategic approaches in Indonesia

**DOI:** 10.1186/s12913-026-14254-2

**Published:** 2026-02-26

**Authors:** Cut Ainul Mardhiyyah, Firda Shafira Nurfadila, Shazia Jamshed, Ida Ayu Andri Parwitha, Dian Ayu Eka Pitaloka, Ivan Surya Pradipta

**Affiliations:** 1 Doctoral Program of Pharmacy, Faculty of Pharmacy, Universitas Padjadjaran, Sumedang, 45363 Indonesia; 2 Department of Pharmacology and Clinical Pharmacy, Faculty of Pharmacy, Universitas Padjadjaran, Sumedang, West Java 45363 Indonesia; 3 Faculty of Pharmacy, Universitas Yayasan Pendidikan Imam Bonjol Majalengka, Cirebon, 45135 Indonesia; 4 Pharmacoepidemiology and Drug Utilization Study Group, Center of Excellence in Higher Education for Pharmaceutical Care Innovation, Universitas Padjadjaran, Sumedang, 45363 Indonesia; 5 Department of Pharmacy Practice, School of Pharmacy, International Medical University, Kuala Lumpur, Malaysia; 6 Department of Clinical and Community Pharmacy, Widya Mandala Surabaya Catholic University, Surabaya, 60112 Surabaya, Indonesia

**Keywords:** Community pharmacist, TBI, TB preventive treatment, Qualitative, Supporting treatment

## Abstract

**Background:**

Community pharmacists (CPs) are accessible healthcare providers with the potential to support care for tuberculosis infection (TBI). However, their role remains limited and poorly integrated into national TB programs. Despite global recognition, no study in Indonesia has explored how CPs can be engaged as direct service providers. This study aims to identify the challenges and develop strategies for involving CPs as direct providers of TB services to improve TB preventive treatment outcomes.

**Methods:**

A qualitative case study was conducted in West Java, Indonesia. Data collection involved group interviews (GIs) and in-depth interviews (IDIs) with providers from community health centers (CHCs) and community pharmacies, as well as with patients with TBI. Participants responded to a proposed collaborative model scenario during the interviews, where CPs would provide direct support for TBI treatment. Data were deductively analyzed using ATLAS.ti version 9, employing a thematic analysis approach guided by the Tailored Implementation for Chronic Diseases (TICD) framework to identify challenges and strategies across seven domains. The findings are reported in accordance with the 32-item COREQ checklist.

**Result:**

A total of 27 participants were enrolled from CHCs and pharmacies, including TB programmers, medical doctors, community pharmacists, and individuals with TBI. We identified several challenges, including the absence of specific guidelines; limited knowledge and skills among CPs; patient preferences; limited professional interaction between CPs and healthcare workers; the lack of an incentive scheme; and insufficient regulatory support from national and local governments. In response to these challenges, various strategies can be implemented, including providing systematic guidelines, improving CPs’ capacity, building a communication system between CPs and related healthcare workers, implementing an incentive scheme, and advocating for regulatory support from national and local governments.

**Conclusion:**

To implement collaborative practice effectively, it is essential to address the identified challenges and, alongside coordinated efforts among all TB stakeholders, develop strategies to establish a sustainable, impactful practice model in real-world settings.

**Supplementary Information:**

The online version contains supplementary material available at 10.1186/s12913-026-14254-2.

## Introduction

Indonesia remains one of the leading contributors to global tuberculosis (TB) cases. According to the Global TB Report 2025, Indonesia is the second-highest country for pulmonary TB cases, representing over 10% of the global TB burden [1]. The TB Indonesia 2025 dashboard indicates a notable increase in TB cases compared to the previous year, with 856,420 new infections in 2024 and an estimated total of 1,090,000 cases [2]. This rise in TB active or newest term TB Disease (TBD) cases elevates the risk of transmission of Mycobacterium tuberculosis and the development of Tuberculosis Infection (TBI), particularly among high-risk populations [3]. Approximately one-quarter of the global population is estimated to have TBI, which remains asymptomatic but may progress to active TB if not appropriately managed [3]. The high prevalence of TBI presents a substantial public health challenge, as undiagnosed and untreated individuals may contribute to future transmissions and hinder TB elimination efforts.

To mitigate the risk of progression from TBI to TBD, individuals with TBI should take TB preventive treatment (TPT) [3]. There are any types of TPT treatment: the first is 3HP (Isoniazid and Rifapentine, taken every week for 3 months), 3H (Isoniazid and Rifampicin, taken every day for 3 months), 6H (Isoniazid, taken every day for 6 months), and 6LFX + E (Levofloxacin and Ethambutol, every week for 6 months for drug resistance TB (DR-TB) patient close contact) [3, 4]. However, the coverage of TPT in Indonesia is alarmingly low, with only 2.4% of the targeted population expected to be reached in 2024 [5]. This result falls significantly short of the national target of 68% [4]. Consequently, this gap will lead to an increasing number of TBD cases, which can further exacerbate the TB burden in Indonesia [4].

Community pharmacists (CPs), who manage pharmacies, play a significant role in addressing TBI cases [6]. They are extensively available across the urban village, sub-districts, and districts, providing convenient locations for TBI management [7]. CPs possess the knowledge needed to provide comprehensive, patient-centered care for TBI, including ensuring medication adherence, managing adverse effects, and supporting TB prevention initiatives [8]. On the other hand, high workload of TB programmers at the community health center (CHC) level was reported in Indonesia, which led to sub-optimal TB services since CHC is a backbone facility for TB services in Indonesia [9–11].

Evidence suggests that CPs can play a supportive role in TB and TBI treatment [6, 12]. A study in New Mexico showed that collaboration between the CPs and the health department achieved a 75% completion rate over a 12-week treatment period [6]. Strengthening the finding, another study in the United States revealed that completion rates were 59% for a 9-month regimen and 67% for a 6-month regimen in a pharmacist-managed clinic on a college campus [12]. Those studies highlight that involving CPs in a collaborative TBI treatment program has not only alleviated the workload for healthcare in CHC but also furnished patients with more accessible treatment options [6, 13, 14].

Despite the promising role for better TB treatment outcomes, CPs’ involvement in TB services in Indonesia remains minimal. A cross-sectional study revealed that only 2% of CPs provided direct TB services within the community in Indonesia [15, 16]. Although the World Health Organization (WHO) and the International Pharmaceutical Federation (FIP) reached a joint understanding in 2011 on the importance of involving pharmacists in TB treatment support, implementation remains constrained [17]. Regrettably, CP’s involvement in TBI management has not been established in Indonesia, underscoring the need for additional research to develop CP programs as direct service providers to support the treatment of individuals with TBI. Given current scientific evidence and the potential role of CPs, this study aimed to explore the challenges and develop strategies for an effective, sustainable collaborative model that involves CPs as direct service providers in TBI treatment.

## Methods

### Study design

This study employed a qualitative case study design. The study was conducted from May 2024 until February 2025 in Bandung City and Cirebon City. Bandung and Cirebon were selected to represent an urban, well-resourced setting and a high-burden TB area, allowing comparative insights into community pharmacist involvement in TBI care. Information was gathered from participants’ perspectives on a case scenario. The scenario outlines a proposed collaborative practice model, developed from findings in our previous literature review, which was published as a separate study [18]. In Indonesia, the District Public–Private Mix (DPPM) TB guidelines state that private pharmacies led by CPs are expected to provide anti-TB medicines, dispense prescriptions from health facilities, provide counseling, support medication adherence monitoring, and collaborate with physicians and TB services [19]. However, in routine practice, private pharmacies remain minimally involved in structured TB treatment monitoring and reporting. Their role is largely limited to dispensing TB medicines—often non-program branded products—based on physicians’ prescriptions [15, 16]. Moreover, private pharmacies are not currently integrated into TPT services under the National TB Program [20], and no standardized operational guidance exists for CPs in TB infection care [15, 16]. This gap underscores the need for a structured model to support feasible and context-appropriate pharmacist engagement in TB control.

**Fig. 1:**
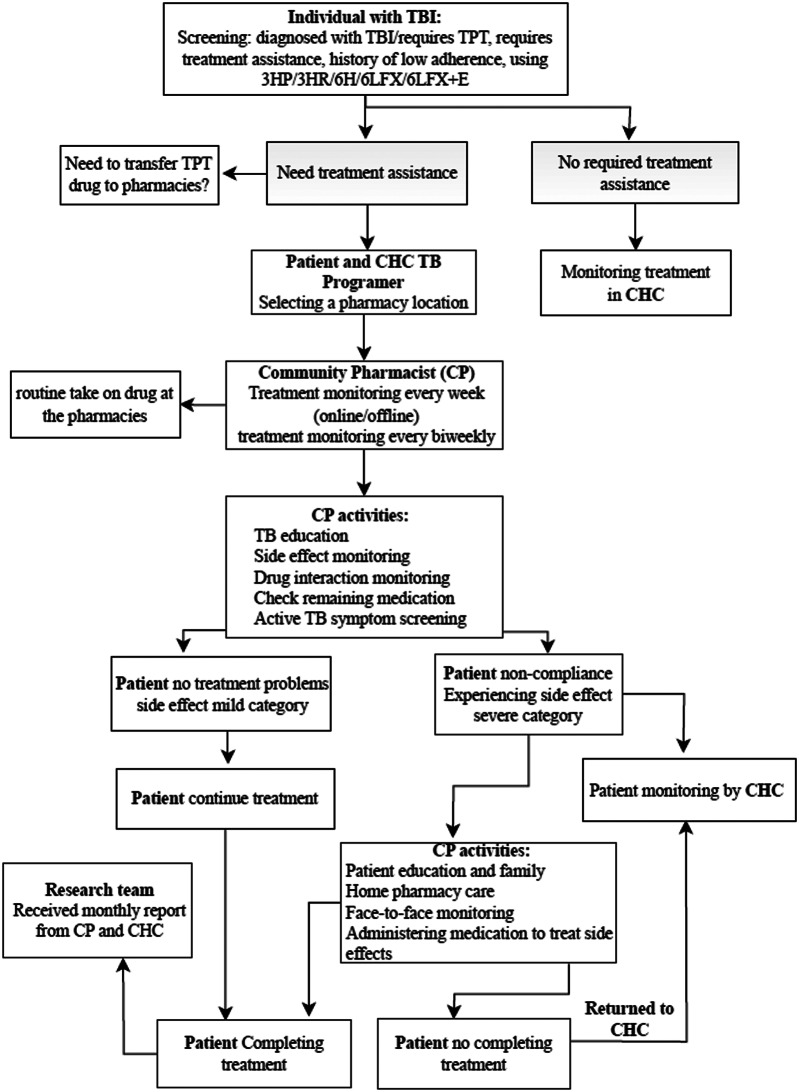
The proposed collaborative TBI care model involving community pharmacists

The proposed CP-assisted treatment model for individuals with TBI is shown in Fig. 1. The proposed model begins with individuals with TBI who receive TPT at CHC and undergo an assessment to determine their need for treatment support. If a patient requires assistance, they can choose a designated pharmacy for pharmacist support. Afterwards, a patient on TPT medication will transfer from CHC to the pharmacy. The TPT medication can be collected every two weeks from the pharmacy, or patients may pick it up weekly or obtain it directly. People Living with Human Immunodeficiency Virus (PLWH) receiving TPT have access to online supervision, or they can arrange alternative monitoring methods with the pharmacist before starting treatment. CPs at pharmacies oversee treatment, ensure medication adherence, monitor adverse effects and potential drug interactions, educate about TB, screen for active TB symptoms, and manage potential drug-related problems (DRPs). Assessments are typically conducted throughout the three- to six-month treatment period, in accordance with guideline protocols, until the patient completes treatment. CPs maintain regular communication with the TB program staff and CHC pharmacists regarding patient treatment and care.

Two researchers conducted data collection to address potential cultural bias. CAM is a female researcher trained in quantitative and qualitative methodologies, currently pursuing a doctoral degree in pharmacology and clinical pharmacy. FSN is a female clinical pharmacist presently enrolled in a master’s program in pharmacoepidemiology, where she focuses on barriers to and strategies for treating TBI. Both researchers had undergone training in TB and qualitative research methods. There was no prior relationship between the researchers and participants to avoid the potential power imbalance that could affect the quality of the interviews and discussions.

The model described in this study is not yet implemented as a routine TB service in Indonesia. It was previously developed by our team with a structured collaborative care scenario to explore implementation challenges and potential strategies. All participants were informed that the study focused on this proposed model, which involves community pharmacists working in collaboration with CHCs to reduce downstream losses after TPT initiation. To our knowledge, this is the first explicitly described model in the Indonesian context with this specific workflow, defined pharmacist role, and structured CHC–CP coordination. Following this formative qualitative phase, the model is being prepared for pilot implementation and evaluation in Indonesia during 2025–2026.

### Research context and setting

Indonesia’s healthcare system includes both public and private facilities. CHCs serve as the primary healthcare providers at the district level and are managed by local government authorities [21]. In Bandung City, 2–3 CHCs are available in every subdistrict, whereas in Cirebon City, CHCs are available in every urban village. Pharmacies, on the other hand, are privately operated healthcare facilities that offer pharmaceutical services, including dispensing medicines and providing clinical pharmacy support. Although TB notifications are increasing, TPT completion rates remain insufficient. In 2023, in West Java Province, including Bandung City and Cirebon City, the TPT completion rate was 68.3%, which remains below the national target of 80%, indicating ongoing losses after treatment initiation [22]. This discrepancy underscores the importance of implementing support strategies after treatment initiation, such as the community pharmacist–assisted approach suggested in this research. They are widely distributed across urban villages and sub-districts under the supervision of the CHC in their respective regions. Each CHC in Indonesia has a TB unit responsible for prevention, detection, diagnosis, treatment, monitoring, education, and reporting. TB services are typically provided by a multidisciplinary team consisting of physicians, nurses (often serving as TB program coordinators), laboratory analysts, and pharmacists [21].

### Study participants

Participants were purposefully selected using criterion-based sampling to ensure relevance to the collaborative TBI care model. They were drawn from different CHC facilities and community pharmacies to capture variation in experiences and service contexts. Eligible participants were those with direct experience or substantial knowledge of TB-related services, challenges, or best practices within their facilities. Participants from CHCs were TB programmers and pharmacists with at least 6 months of TB service-delivery experience, and they worked at CHCs that had prior experience managing TBI cases.

The patients identified were those undergoing TPT or who had completed TPT. The CPs we identified are pharmacy workers and pharmacy owners. Since the successful implementation of pharmacist-led TBI services depends on multisectoral collaboration, we also involved key stakeholders engaged in TB program planning and oversight. This included the heads of CHCs, the district TB Supervisor, and representatives from the Indonesian Pharmacists Association. Participants were contacted directly through formal invitations and coordination with the District Health Office and the Executive Committee of the Indonesian Pharmacists Association. The final number of participants was determined by data saturation, which was reached when no new themes or insights emerged from the interviews.

### Data collection

We provided the selected participant with a written consent form before the interview. We conducted in-depth interviews (IDIs) at times convenient for the participants. Each interview began with general questions in Indonesian, after which the interviewer explored the information based on previously established interview guidance [23]. Field notes were made during and after the interview. All interviews were recorded, using audio-only for in-person interviews and audio-visual recording for online interviews. This approach was guided by pragmatic and ethical considerations. For in-person interviews, audio recording was used to prioritize participants’ comfort and privacy, minimizing perceived intrusiveness while adequately capturing verbal data required for thematic analysis. For online interviews, audio-visual recording (with consent) facilitated rapport-building and smoother interaction on digital platforms, while allowing limited observation of non-verbal cues (e.g., facial expressions) that could support interpretation. To ensure data consistency across modes, we used the same semi-structured interview guide, probing strategies, and interviewer training. Field notes and reflexive memos were maintained for all interviews. Transcription, coding, and theme development followed identical analytical procedures regardless of interview format. The general questions were, “Can the proposed collaborative TBI care model with community pharmacists be applied in the field?” and “What are the potential challenges to applying the model in a field study?” According to the guide in Supplementary File 1, the interview followed several steps.

### Data processing and analysis

Transcripts were transferred to Atlas. ti version 9 software for data analysis. Deductive data analysis was conducted in several steps: introduction, identification of a thematic framework, codification, and interpretation [24]. CAM coded the transcript data, classified them into themes/subthemes, and discussed them with FSN. Any disagreements in data analysis were resolved by considering both transcripts and field notes. All transcripts were analyzed in Indonesian. After coding, selected excerpts included in the manuscript were translated into English by two bilingual researchers (CAM and FSN). The translations were verified against the original Indonesian quotes, and any differences were addressed through discussion; a third researcher (IAAP) reviewed a subset of translated quotes for accuracy. Other researchers (ISP and DAE) reviewed the final draft codes, themes, and subthemes. We identified challenges to 7 themes based on the Tailored Implementation for Chronic Diseases (TICD) framework [25]. Namely: (1) guideline factors, (2) individual pharmacist, (3) patient factors, (4) professional interactions, (5) incentives and resources, (6) capacity organizational changes, and (7) social, political, and legal factors. TICD was selected because it provides a comprehensive yet pragmatic structure that integrates behavioral, interpersonal, organizational, and policy-level determinants specific to chronic disease management. Unlike the Consolidated Framework for Implementation Research (CFIR), which is broader in scope, and Theoretical Domain Framework (TDF), which focuses mainly on individual behavioral change, TICD facilitates direct tailoring of implementation strategies based on multi-level determinants relevant to chronic disease care, such as tuberculosis.

### Credibility of information

We followed the Lincoln and Guba Framework, which outlines a series of techniques for qualitative research to establish the credibility of the information in this study. This framework indicates all four criteria of dependability, transferability, and confirmability to establish trustworthiness [23]. We combined data and researcher triangulation to increase the trustworthiness of the information obtained from participants. Essential information from a participant was corroborated by other participants in the context to ensure its credibility. Researcher triangulation was conducted to verify the findings among researchers. We used the COREQ-32 checklist to provide transparent and systematic reporting in a qualitative study [26]. To maintain the study’s integrity and confidentiality, data were collected in controlled settings where only participants and researchers were present. This ensured a safe and open environment, allowing participants to express their thoughts without external influence.

## Result

### Characteristics of study participants

Of 32 invited participants, five declined. One TB programmer from a CHC opted out because their facility lacks TBI services. Three potential patient participants declined due to scheduling conflicts, and one was out of town. Notably, no participants withdrew after initially consenting to participate. This study included 27 participants: 17 healthcare professionals responsible for TB management at CHC, two CPs working in pharmacies, two representatives from the Indonesian Pharmacists Association, and six individuals with TBI. The participants were majority female (77.8%) and between 36 and 45 years old (59%). We conducted IDIs with 23 participants, interviewing four of them twice to confirm their initial responses and address follow-up questions. Additionally, two group interviews (GIs) were conducted at participants’ request, even though they had initially agreed to IDIs; the GIs included four participants who were not previously engaged in the IDIs. GIs were conducted 2 times, with 2 participants each. Participant characteristics are summarized in Table 1.

**Table 1:** Characteristics of participants (*N* = 27)

Characteristic	Number
**Gender**	
Male	6 (22.2)
Female	21 (77.8)
**Age (year)**	
26–35	9 (28.6)
36–45	16 (59)
46–55	2 (7.4)
**Subject background**	
CHC TB Programmer	5 (18.5)
District Health TB Supervisor	2 (7.4)
CHC Pharmacist	4 (14.8)
Head of CHC	3 (11.1)
CHC Doctor	3 (11.1)
Community Pharmacist	2 (7.4)
Professional Organization of Pharmacist	2 (7.4)
Individuals with TBI	6 (8.6)
**Data collection method**	
IDI only	23 (85.2)
Group interview only	4 (14.8)
**Average interview duration (min-max) in minutes**	
IDI (20–74)	48
Group interview (62–84)	73
**Interview location**	
CHC	14 (51.9)
District health office	1 (3.7)
Pharmacies	1 (3.7)
Online meeting	11 (40.7)

During data collection, no participants participated in both methods we used (IDI and GI). All discussions were conducted exclusively in Indonesian, as all participants were proficient in spoken and written communication, ensuring clarity and uniform understanding throughout the study. No other local languages were used, eliminating potential barriers to effective data collection and interpretation.

Participants generally responded positively and optimistically to the proposed collaborative practice model. CPs expressed enthusiasm about expanding their role in supporting TBI patients, while emphasizing the importance of adequate training to ensure implementation readiness. TBI patients also welcomed pharmacists’ involvement as treatment partners, particularly when the approach was communicative and educational. At the district/city TB program level, one participant expressed hesitation, noting that integrating CPs into TBI care is relatively new and that not all regulators or policymakers fully understand its operational details. Nonetheless, other healthcare professionals indicated support, provided that clear guidance and evidence of CPs’ concrete contributions are available.

### Challenges to engage CPs in TBI care

**Fig. 2:**
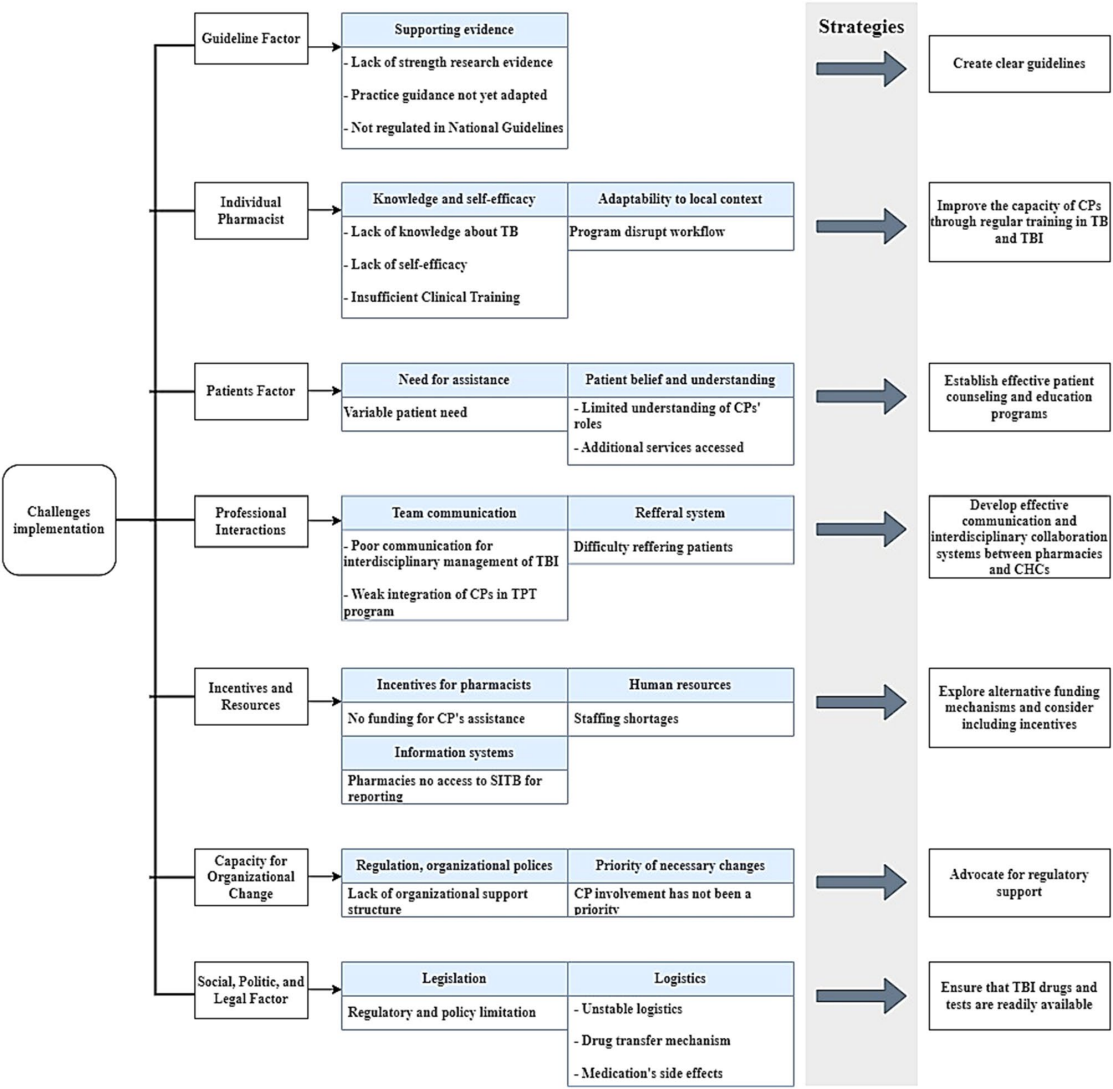
Challenges and strategies for CPs as direct service providers for individuals with TBI

The meaning unit was coded and grouped into relevant themes and subthemes according to the TICD framework; the resulting list is provided in Supplementary file 3. The researchers analyzed participant responses to identify recurring patterns. The coding process is provided in Supplementary 2. The results of the challenge identification are presented in Fig. 2.

#### Guideline factor

The highlighted guiding factor encompasses the subtheme of supporting evidence, indicating a lack of strength of research evidence in Indonesia regarding CPs’ involvement in monitoring individuals with TBI, which is currently limited to CHC and government hospitals [27].

*There are no studies and guidelines for community pharmacists to assist patient TBI at this time.* Female, TB Programmer CHC.

The practice modules offered must adapt to the health facilities during implementation. However, guidelines for pharmaceutical services for TB patients were developed and issued by the Ministry of Health Indonesia in 2024 [27]. Their implementation in the field, particularly at the community pharmacy level, remains suboptimal. The guidelines also do not address regulations and best practices for strengthening CPs’ proposed model of TBI treatment assistance.

*Since each pharmacy and possibly CHC has an internal policy in practice*,* the guidelines and modules must be adapted to the local context in each pharmacy and CH*C. Female, pharmacist professional organization.

The practice of CPs assisting patients with TBI has not been regulated in the National Guidelines for TB Control [5]. Currently, the existing role involves treatment assistance provided by trained cadres and TB volunteers, but this support is limited to active TB patients.

*The program offered has not been regulated in the National TB guidelines*,* especially related to the mechanism of transferring drugs to pharmacies*,* which requires a policy from the Ministry of Health.* Female, District Health TB Supervisor.

#### Individual pharmacist

A lack of knowledge, skills, and self-efficacy poses a challenge for pharmacists in handling TB patients. The issue is that they do not treat patients with TBI or active TB, despite rules on private sector involvement in TB treatment [28].

*For example*,* if we had prior knowledge about TB*,* we would feel confident. Because we lack knowledge*,* so yes*,* we are also not confident to handle it*. Female, CP.

The relationship between CPs’ knowledge, skills, and confidence in managing patients with active TB and TBI, as well as their habits—which tend to focus more on logistical or sales activities—results in a lower likelihood of being influenced by government programs implemented at the CHC level. The CHC and the local health office do not guide this particular CP.

CPs lack specialized clinical training in TBI management and may feel inadequately prepared to handle TBI treatment monitoring, adverse drug reaction management, and logistics.

*We have not had any special training in handling TBI patients*,* and we have never heard that there is a term*,* Latent TB*. Female, CP.

This case demonstrates the program’s suitability for the local context, enabling local program implementers to implement it. The challenge concerns CPs in pharmacies who perceive that this practice is disrupting their current workflow.

*I am the only pharmacist in the pharmacies*,* and I have other responsibilities outside of this job*. Female, CP.

#### Patient factors

The challenge is to analyze the support model patients need, as assistance must be tailored to individual circumstances to ensure effective engagement. Patients who decline support could either be confident in managing their treatment independently or might be at greater risk of disengaging from care.

*In terms of assistance*,* I have observed that one patient appreciates receiving a daily message on WhatsApp*,* while another patient prefers not to receive such messages.* Female, TB Programmer.

Another challenge is the lack of awareness and recognition of CPs among patients and communities, which limits their role in patient education and TB treatment support.

*Yes*,* when I go to the pharmacies*,* I find him*,* not the same pharmacist*,* so I don’t know him.* Men with TBI.

Other challenges identified are that the program CPs to support the treatment of individuals with TBI will increase the services patients access, increasing patient costs and time.

*The program*,* by directly referring TBI patients to the pharmacies*,* will increase the number of services that must be accessed*,* as well as transportation costs*,* service fees*,* and others*. Female, District Health TB Supervisor.

#### Healthcare professional interactions

The challenge faced by health professionals is the lack of a network communication system between CHC and CP, particularly regarding the TB program. Despite being a part of CHC’s coaching network, CHC’s programs rarely impact CPs in pharmacies.

*The pharmacies have not been exposed to any CHC program so far.* Female, CP.

Weaknesses in integrating pharmacists to assist with TBI management at both the local and national levels have hindered coordinated care. The lack of pharmacist involvement in TB service delivery hinders synergy across health sectors, reducing the effectiveness of overall TB patient management.

*Because there is no regulation*,* and it is not yet possible to integrate CP in TBI treatment*,* it is also worrying that they have not been able to manage patients properly*,* especially patients who are lost to follow-up.* Female, District Health TB Supervisor.

One obstacle that may arise is the ability of TB personnel in CHC to refer patients, communicate with them, and convince them of the benefits of CP’s treatment assistance.

*CHC will find it hard to refer patients to get the right education and reasons to be referred due to the constraints of patients who do not want to visit two places.* Female, District Health TB Supervisor.

Meanwhile, these challenges contradict the results of our interviews with patients. All patients we interviewed agreed that it would be highly beneficial to have TPT medication pickup available at the nearest pharmacies. They mentioned that this approach would save time, reduce transportation costs, and make it easier to adhere to the treatment schedule, especially for those living far from health facilities.

*Yes*,* it’s good if you pick up the medicine at the pharmacies*,* so you don’t have to queue and take a long time to pick up the medicine at the CHC*. Female, TBI Patient.

#### Incentives and resources

Currently, there is no funding system for TB treatment assistance by CPs for both active TB patients and TBI. Only TB cadre assistance is provided in the urban village for active TB patients, funded by the Global Fund.

*Well*,* the funded transportation is only active patient assistance by cadres.* Female, CHC TB Programmer.

We have successfully identified resource challenges. In fact, only some pharmacies have more than one CP, so if the CPs own the pharmacy, they will be busier with management and profit.

*I am the only pharmacist in the pharmacy*,* because it is also a small pharmacy*,* so I take care of everything myself.* Female, CP.

The information system challenge is access to online reporting, specifically the “Sistem Informasi Tuberkulosis” (SITB) app, which allows users to report all TB-related activities in Indonesia. There is no regulation allowing pharmacies to self-report in this system; therefore, during implementation, pharmacies must have reporting tools that can be easily transferred to the CHC level.

*Access to SITB is limited to CHC and hospitals*. *This program will be constrained by the requirement to record and report through SITB*,* as patient referrals can only be made to health facilities registered with SITB. Currently*,* there is no rule governing whether pharmacies outside the CHC can access SITB.* Female, District Health TB Programmer.

#### Capacity for organizational changes

This theme was identified from the perspective of professional pharmacists from the organizations overseeing CP, the District Health Office, Community Health Centers, and pharmacies. Currently, there are no formal guidelines, incentives, or recognition of CP for TB management activities from these organizations.

*Because there has been no circular or warning from professional organizations that the pharmacies must be involved.* Female, Professional Organization of Pharmacists.

CPs are not yet aware that TB management programs can involve them in pharmacies. Some believe the government already handles TB treatment, and they have not been exposed to regulations regarding private sector involvement in TB management.

*Yes*,* because we also don’t know what programs are at the CHC*,* including the TB program.* Female, CP.

#### Social, political, and legal factors

As with the model implementation guidelines, government regulations and policies do not explicitly mention that CPs in pharmacies assist individuals with TBI treatment, as this program is an innovation.

*There is no policy allowing CPs to assist in the treatment of TBI patients; however*,* assistance is available for cadres*,* NGOs*,* and other health workers.* Female, District Health TB Programmer.

Unstable logistics are obstacles to TPT services [29] and the sustainability of the CP to individuals with the TBI assistance program. The new 3HP therapy regimen, introduced in 2022, experienced instability in Indonesia in early 2023 and continued into 2024. Unstable logistical problems will hinder the sustainability of the CP program in assisting individuals with TBI who are given TPT.

*The logistics—that’s the obstacle*,* that’s why I don’t dare to promote TPT anymore; if the logistics don’t exist*,* we are ashamed of the patient.* Female, CHC TB Programmer.

Offering a program to transfer the TPT drug to pharmacies posed an initial challenge, as the drug had to be transported from the CHC to the pharmacies. Still, this program ensured that pharmacies could provide TPT drug services free of charge, rather than patients having to queue at the CHC.

*Yes*,* will CP take the TPT drug from CHC? Alternatively*,* the patient might bring the TPT drug from the CHC to the pharmacies*,* which is also confusing. Don’t let the patient come here later. The medicine is not in the pharmacies.* Female, pharmacist professional organization.

Another problem is the medication’s side effects, whether the patient gets the drug for free at the pharmacies or has to go to the CHC to get medicine to overcome complaints after taking the TPT drug. That is the challenge of transferring drugs to pharmacies; therefore, regulations from local governments, especially the health office, are necessary, or management must be included in the program guidelines.

If the patient experiences side effects that require medication, can the medicine be obtained free of charge at the pharmacy? Therefore, we recommend that the CHC distribute the medication to the pharmacies to manage TPT side effects. Female, Pharmacist Professional Organization.

### Strategies to engage CPs in TBI care

We have identified several responses that discuss and address the strategies proposed by participants, providing insights into their practical application in the field.

Participants stated that the program must be designed with a good communication system in place. This includes ensuring smooth reporting of feedback on CPs’ activities.

*There is also communication from CHC: Does the CP really supervise the patient? And the medicine is given to the patient to take. So*,* later*,* let’s get feedback on the report every two weeks. If this program runs well*,* it’s possible to do this every 3 months.* Female, CHC TB Programmer.

In terms of patient factors, to improve understanding of TPT, patients need to receive clearer information about TPT and the proposed program, and patients need to be more aware of the role of CPs in accompanying them.

*The patient must get clearer information about TPT*,* for example*,* states that the medicine prevents active TB*,* but there are side effects. If it is treated late*,* what will happen? CP also explained it.* Men, TBI Patient.​​.

The importance of method monitoring with patient preference, or an initial offer, needs to be emphasized.

*Evaluation by CP is ok. This is every two weeks*,* right? If it’s weekly*,* that’s fine. If the CP comes to your home*,* that’s more likely. Or*,* it could be offered. Whether the patient comes*,* the CP comes*,* or they can do it online is entirely possible. It’s possible*,* it depends on the agreement with the patient.* Female, CHC TB Programmer.

It is also necessary to involve cadres, NGOs, and peers to introduce the TPT program and emphasize the importance of completing it.

*So it is necessary to have support from TB cadres and NGOs to really help PWLH to accept and complete TPT treatment*,* as well as peer support*, Men, TBI Patient.​​.

During this program, only patients experiencing symptoms will come to the CHC to check their condition and consult with the CHC doctor; the rest will be handled by the CP at the pharmacies.

*So*,* only those experiencing symptoms will be referred to the CHC. So*,* if something like this happens*,* it’s fine; the medication will be transferred under a CP’s supervision*. Female, CHC TB Programmer.

In this monitoring program, participants suggested that it be carried out by pharmacies that collaborate with public health insurance.

*Maybe you could involve pharmacies that have collaborated with public health insurance coverage. Pharmacies that have an MoU with public health insurance coverage have a high likelihood of engaging CPs to conduct monitoring.* Women, CP.

This program can be implemented if there is funding from WHO for pharmacist incentives and operational costs of CHCs and pharmacies.

*It’s possible if there is program funding from WHO for incentives and operational costs*. Female, Head of CHC.

There is a need for cooperation among the professional organizations of health workers involved in this program, in order to receive a reward in the form of an PCS point.

*Yes*,* there’s a collaboration between the pharmacist organization and the nurse organization*,* so we can get a PCS to the “Plataran sehat”.* Female, CHC Pharmacist.

This implementation program needs to socialize with pharmacy owners to understand the benefits for their pharmacies, even if some CPs are also pharmacy owners.

*Yes*,* the pharmacy owner also needs to be socialized to raise awareness and benefit from the program.* Female, CP.

In some cases of TPT logistics shortages, one strategy is to borrow from CHCs if TPT drugs are out of stock at one CHC but still available at another.

*We’re smart: if there’s a shortage*,* we can borrow the system from other CHCs so we can still meet needs if the Health Department really doesn’t have TPT drugs.* Female, CHC Pharmacist.

There is a need for logistical certainty from the central/ministerial level for the sustainability of the TPT program, which also has an impact on the CP involvement program in TBI care.

*From above*,* if we want the mandatory TPT*,* so that logistics must be fulfilled*,* we will implement it*. Man, Head of CHC.

This strategy will be strengthened by recommendations from researchers in the discussion section, which are grounded in empirical findings from previous studies and data analyses related to TBI, and supported by a scientific, evidence-based approach.

## Discussion

This study was purposefully designed to identify implementation challenges in the early stages of developing a collaborative TBI care model, as perceived by healthcare professionals and patients. Accordingly, the interview guide and deductive analytical approach were problem-oriented, limiting systematic exploration of facilitator factors. Although some positive elements (e.g., patients’ willingness to collect TPT medicines at nearby pharmacies, longer pharmacy hours, and the DPPM policy) emerged in interviews, they did not reach saturation and were not developed as standalone themes. We develop strategies to mitigate the identified challenges, organizing them into seven themes as shown in Fig. 2.

The model’s design limits pharmacists’ responsibilities to protocol-based treatment-support tasks, including adherence reinforcement, education, monitoring of potential adverse drug reactions, and referral of symptomatic patients, while diagnostic and prescribing authority remain at CHCs. This task-sharing approach fits within pharmacists’ established scope in medication management and chronic disease support, which strengthens practical feasibility in private pharmacy. In this context, CPs are envisioned as protocol-based treatment-support partners within the TB program, rather than independent TB care providers.

### Strategy 1: Create clear guidelines

A major issue identified is the lack of government-issued guidelines outlining CPs’ role in TBI treatment. Pharmaceutical care guidelines for TB treatment were introduced in 2005 and are widely used by pharmaceutical personnel [30]. It is not strong enough to serve as a guideline for CP in handling TBI. However, the guideline states that pharmacies should provide information services and be willing to supervise treatment for TB patients. In the 2016 guidelines for pharmaceutical services in pharmacies, drug information service activities include providing patient education and counseling, including for patients receiving long-term therapy or managing chronic diseases such as TB [31].

In 2023, the Ministry of Health introduced a Distance Training Module on TB Control Program Management for Pharmacists in Health Care Facilities [27]. However, this module has not been widely applied, particularly among pharmacists in private-sector pharmaceutical services. This situation reflects a gap between central-level policies and implementation at the public health service level. This lack of implementation can be attributed to multiple factors, including inadequate outreach and training, as well as the absence of an effective monitoring and evaluation system for implementing these guidelines. Consequently, pharmaceutical services for TB patients in pharmacies have not met expected standards. To improve access to and influence over TB care, the government should promote and implement these guidelines, particularly for CPs. Given that this is a new initiative, it is essential to disseminate research information from other settings that substantiates the successful execution of such programs.

### Strategy 2: Improve the capacity of CPs through regular training in TB and TBI

In dealing with problems related to the lack of knowledge, attitudes, skills, and practices of CPs in TBI services, the proposed strategy is to improve the capacity of CPs through regular training in TB and TBI [32, 33], at least once every 6 months or when there is a latest guideline update; training can also foster confidence in handling patients [34–36]. In the 2009 TB Control Guidelines, it is stated that training is one of the efforts to improve officers’ knowledge, attitudes, and skills to improve their quality and performance in TB services [37]. Training in the TB program can include clinical aspects, program management aspects, and initial training in basic DOTS implementation [38].

TBI treatment assistance training programs can be standardized across the public and private sectors, driven by the DPPM network, with the implementation of a national curriculum, competency-based training, and integrated cross-sector monitoring and evaluation systems [39]. Training modules covering TBI screening, TPT administration, effective patient communication, and medication logistics management are in accordance with national regulations and guidelines. Training methods include classroom instruction, field practice, e-learning, and psychosocial support. Evaluation is conducted through reflection, group discussions, and reporting of participants’ competency status/scores. Evaluation using self-assessment, pre- and post-tests, and PCS submissions can be included in quality monitoring. Standardization is also carried out through periodic assessment by the Ministry of Health or District/City Health Offices, with feedback from the community and private institutions [5]. However, due to a lack of national guidelines, health education institutions must take the initiative to prepare CPs for active engagement in patient services. CPs can serve as treatment advocates, providing education, monitoring, and medication evaluation in accordance with pharmaceutical care principles [40]. Previous studies in the USA have demonstrated that pharmacist involvement in TB patient management enhances treatment success to 94% [41].

### Strategy 3: Establish effective patient counseling and education programs

The patient-related challenges are often interrelated, and we identified those that require treatment assistance. In the TBI 2020 technical instructions, it is stated that in choosing a monitoring method, we can use the directly observed treatment (DOT) or self-administered treatment (SAT) method, which is adjusted to the local context, patient preferences, and/or other considerations such as the risk of developing into severe TB disease [3, 4]. In addition to overcoming the lack of patient information about the role of CPs in TB management, CPs must introduce themselves as pharmaceutical personnel who play a role in handling TB by educating patients and increasing the role of the head of the family in making decisions [42]. Studies in Indonesia have shown that although pharmacy personnel generally have good knowledge and a positive attitude towards TB detection and treatment, their actual practice is limited [15, 16] so that it is not widely known to the public. With this education, CPs can improve patients’ confidence in starting and completing TPT treatment, increase patient adherence, and manage side effects patients feel by managing therapy guidelines [33].

The stigma experienced by individuals receiving TPT medications may resemble that faced by people collecting TB treatment in pharmacies and should therefore be considered when designing pharmacy-based TPT services. Evidence suggests that stigma can cause patients to feel embarrassed, fearful, and hesitant to be seen taking TB medications in public or semi-public settings [43], including pharmacies. Such perceptions may reduce patients’ willingness to disclose concerns, undermine their confidence, and negatively affect treatment adherence [44].​ Research in China further indicates that patients who anticipate being judged by others at pharmacies may limit social interactions, delay medication pickup, or avoid visits altogether. This is particularly relevant for TPT, which is often provided to individuals who appear “healthy” but are perceived as being “at risk for TB,” potentially heightening concerns about social labeling [45].

The collaborative care model has limitations, relying on individuals with TBI being aware of their condition and actively seeking TPT to protect themselves from developing active TB. Patient awareness and initiative in seeking TPT are key factors in reducing TB cases by addressing social stigma. The success of this model may depend on patient engagement with their TBI status, which can limit its effectiveness if awareness or access is low. Therefore, strategies to improve TBI screening and patient education are needed to address these limitations.

### Strategy 4: Develop effective communication and interdisciplinary collaboration systems between pharmacies and CHCs

Developing effective communication and interdisciplinary collaboration systems between pharmacies and CHCs can help overcome limited professional interaction [46]. As stated in the WHO guidelines on public-private mix, it needs to involve the government and the private sector in actively addressing TB [28]. Interdisciplinary collaboration in healthcare involves problem-focused processes, information sharing, and teamwork among healthcare professionals [47]. Effective collaboration requires interprofessional education, role awareness, and interpersonal skills [47–49]. A mentoring program delivered through home visits by networked CPs helps address gaps in the current referral system, which remains poorly integrated between private pharmacies and CHCs. While CHCs serve as frontline TB units, they continue to face challenges in coordinating referrals with community pharmacies. Home visits by CPs enable direct monitoring, adherence support, and DOT provision without increasing CHC staff workload or imposing additional costs on patients who obtain medications from pharmacies. Evidence indicates that CPs can support referrals, provide education, monitor side effects, and offer counseling for TB treatment, with approximately 70% expressing willingness to supervise TB-DOT. However, home visits entail higher operational costs—including transportation, staff time, and logistical needs—that the current system does not cover. Decision-makers must weigh these costs against the benefits, such as improved TPT adherence and reductions in active TB cases. Potential solutions, such as appropriate incentives or digital tools, may help offset these expenses [50]. Strategies that can be used to overcome the constraints of CP’s limitations in accessing reports at SITB are to collect the same information needed by SITB in the same format and send it to the TB program manager at CHC to be included in SITB, or to campaign for pilot programs and mutual agreements or Memorandum of Understanding (MoU) that allow CPs to access SITB directly [49].

### Strategy 5: Explore alternative funding mechanisms and consider including incentives

Financial constraints present an additional challenge for CP in delivering direct services to individuals with TBI. Several incentive mechanisms may encourage pharmacist engagement in TPT provision, including financial incentives, opportunities for professional development (e.g., training, seminars, and additional Professional Credit System (PCS) credits), and formal recognition from professional organizations or the government. Alternative funding approaches, such as public health insurance coverage or pharmacy-based incentive schemes, may also serve as remuneration strategies to compensate pharmacists for the time and services provided in caring for individuals with TBI [51, 52]. A study in Brazil demonstrates that cash incentives can effectively increase participation among health workers and other cadres in TB programs, suggesting that similar approaches could be applied to CP [53].

From an implementation perspective, pharmacists involved in TB programs could be eligible to receive PCS for recertification or renewal of their practice licenses, similar to the 5 PCS per year allocated to doctors and nurses actively participating in TB initiatives. Additional forms of professional recognition may include charters or certificates issued by the Indonesian Pharmacists Association, public acknowledgement of outstanding contributions by pharmacists to TB programs, and awards or invitations to participate in national health forums from government or health-sector stakeholders. Opportunities to join TB incubator programs or serve as community-level coaches may further enhance professional exposure and reputation.

### Strategy 6: Advocate for regulatory support

Indonesian pharmacist professional organizations developed an innovative “guest of pharmacist” program for World Pharmacist Day 2024 [54]. The program aims to expand pharmacists’ role in TB screening and education through community counseling and to raise public awareness of TB eradication in Indonesia. However, the program has not been implemented fully in Indonesia due to a lack of information for pharmacists, and it does not include the CP program in monitoring individuals with TBI. Pharmacist professional organizations must be more active in promoting TB programs to ensure they are implemented optimally throughout Indonesia.

### Strategy 7: Ensure that TBI drugs and tests are readily available

Participants perceived that the method of transferring patients to pharmacies increased the number of services accessed by patients, which led to an increase in transportation costs and time required by patients [55]. Even though this program could cut patient services, it led to lost time due to queuing at the CHC during working hours. This program will help patients reduce time and financial barriers to CHC visits, which often lead to missed TBI screening follow-ups and treatment discontinuation. Moreover, logistical barriers further complicate access to care; the central and regional governments must ensure the availability of TPT drug logistics. The provision of TPT drugs cannot be separated from the cooperation between CHC and pharmacies to support individuals with TBI through the CP’s assistance program and to increase the number of patients who receive TPT.

Given limited drug and diagnostic TBI availability, TB high-burden countries (HBCs) may need to prioritize TBI screening for high-risk groups. Therefore, ensuring the availability of TBI diagnostics and medications must be a policy priority. Adjustments to legal frameworks and technical protocols are crucial to improving support for individuals with TBI and accelerating TB elimination efforts in Indonesia. The Government of Indonesia has implemented a TB Service Network in DPPM Health Facilities to strengthen TB services through collaboration [19]. This policy mandates that TB services be provided by a team jointly formed by the district/city health office and health service facilities, both public and private, with support from professional organizations at the district/city level. The DPPM network aims to expand TB services and ensure they meet national standards for service delivery, recording, and reporting. The TB DPPM team comprises representatives from District/City Health Offices (covering disease prevention, health services, planning, etc.), government and private hospitals, CHCs and other primary health care facilities, professional organizations, pharmacist associations, laboratories, and pharmacies [19]. TB control is not only a health issue but also requires cross-sectoral collaboration, given that many individuals with TBI are either reluctant to start treatment or discontinue therapy due to side effects, stigma, lack of information, and non-health-related barriers [10]. A high treatment failure rate could hinder Indonesia’s progress toward Sustainable Development Goals (SDGs) and slow improvements in the Human Development Index (HDI) [56].

### Strengths and limitations

This study has several limitations that should be acknowledged. First, the findings were analyzed based on the occurrence of specific codes in the participant data. At the same time, this approach is commonly used in qualitative research; further quantitative studies are needed to generalize the results to the broader population. Second, the barriers identified in this study may not be universally applicable, as differences in healthcare systems, socio-economic conditions, cultural norms, and political contexts may influence the implementation of TBI treatment. Third, although we included interviews with leaders of pharmacist professional organizations to provide additional context, further studies are needed to comprehensively explore workload implications and understand the real conditions for implementation. Fourth, CPs are unevenly distributed across Indonesia. While our study sites are in areas with relatively easy access to CPs, many regions—especially in eastern Indonesia and remote districts outside Java—have few CP-led pharmacies. This structural imbalance could limit the feasibility and transferability of our model in areas with a limited CP workforce.

Regardless of these limitations, our study has several strength, the study used several strong methods to make sure its results are trustworthy and accurate: including a various of participants based on different traits and locations, keeping data collection consistent whether through in-person or online interviews (which participants agreed to), using techniques to check and confirm the data, such as looking for patterns, finding common themes, and making sure enough data was collected, and having ongoing discussions among the research team to confirm the findings are correct and make sense. The diversity of participants enabled a comprehensive exploration of TBI management from multiple perspectives, including frontline healthcare providers, pharmaceutical professionals, and patients directly experiencing TBI treatment.

## Conclusion

This study examines the challenges of involving CPs in supporting TBI treatment. The study also highlighted the need to implement several strategies to tackle these challenges. These include creating clear guidelines, enhancing CPs’ skills, establishing effective patient counseling and education programs, improving communication between CHCs and CPs, providing incentives, advocating for regulatory support, and ensuring that TBI drugs and tests are readily available. By implementing these strategies, CPs can play a more active role in TBI treatment, ultimately contributing to the elimination of TB in Indonesia.

## Supplementary Information

Below is the link to the electronic supplementary material.

Supplementary Material 1

Supplementary Material 2

Supplementary Material 3

## Data Availability

Due to ethical considerations and the need to protect participant privacy, the data supporting this study are not publicly available. Further information may be provided upon reasonable request, subject to appropriate institutional approvals.

## Abbreviations

CHC: Community Health Center
COREQ: Consolidated Criteria for Reporting Qualitative Studies
CP: Community Pharmacist
DPPM: District-Based Public-Private Mix
DOT: Directly observed treatment
DRP: Drug-related problems
FIP: International Pharmaceutical Federation
GI: Group Interview
HIV: Human Immunodeficiency Virus
IDI: In-depth Interview
PCS: Profession Credit System
PLWH: People Living with Human Immunodeficiency Virus
TBI: Tuberculosis Infection
TBD: Tuberculosis Disease
SAT: Self-administered treatment
SDGs: Sustainable Development Goals
TB: Tuberculosis
TPT: Tuberculosis Preventive Treatment
WHO: World Health Organization

## References

[1] WHO. Global tuberculosis report 2025. WHO; 2025.

[2] WHO. Global tuberculosis report 2024. WHO; 2024.

[3] WHO. Latent tuberculosis infection. Clinical tuberculosis: a practical handbook. 2015.

[4] Kemenkes RI. Penanganan infeksi TB laten. Kementerian kesehatan republik Indonesia. 2020. https://repository.kemkes.go.id/book/73. Accessed 12 February 2025.

[5] Kemenkes RI. Strategi Nasional Penanggulangan Tuberkulosis di Indonesia 2020–2024. Kementerian Kesehatan Republik Indonesia. 2021. https://repository.kemkes.go.id/book/567. Accessed 12 February 2025.

[6] Jakeman B, Logothetis SJ, Roberts MH, Bachyrycz A, Fortune D, Borrego ME, et al. Addressing latent tuberculosis infection treatment through a collaborative care model with community pharmacies and a health department. Prev Chronic Dis. 2020;17:1–9. 10.5888/pcd17.190263. 10.5888/pcd17.190263 PMC7021458 32053480

[7] Traynor K. Pharmacies successfully manage short-course tuberculosis treatment. Am J Health Syst Pharm. 2020;77(14):1093–4. 10.1093/ajhp/zxaa139. 32548614 10.1093/ajhp/zxaa139

[8] Wong YJ, Thum CC, Ng KY, Lee SWH. Engaging community pharmacists in tuberculosis-directly observed treatment: a mixed-methods study. Prim Health Care Res Dev. 2023;24. 10.1017/S1463423623000105. 10.1017/S1463423623000105 PMC10050952 36946302

[9] Pradipta IS, Idrus LR, Probandari A, Puspitasari IM, Santoso P, Alffenaar JWC, et al. Barriers to Optimal Tuberculosis Treatment Services at Community Health Centers: A Qualitative Study from a High Prevalence Tuberculosis Country. Front Pharmacol. 2022;13(March):1–12. 10.3389/fphar.2022.857783. 10.3389/fphar.2022.857783 PMC8990794 35401200

[10] Pradipta IS, Idrus LR, Probandari A, Lestari BW, Diantini A, Alffenaar JWC, et al. Barriers and strategies to successful tuberculosis treatment in a high-burden tuberculosis setting: a qualitative study from the patient’s perspective. BMC Public Health. 2021;21(1):1–12. 10.1186/s12889-021-12005-y. 34670527 10.1186/s12889-021-12005-y PMC8529853

[11] Akwaowo C, Umoh V, Umoh I, Usoroh E, Motilewa O, Ekpin V, et al. Effectiveness of providing cash incentives and training to community health workers on active case finding for tuberculosis in Nigeria: a cluster-randomized control trial. F1000Res. 2024;10:1154. 10.12688/f1000research.53822.2.

[12] Hess K, Goad J, Wu J, Johnson K. Isoniazid completion rates for latent tuberculosis infection among college students managed by a community pharmacist. J Am Pharm Assoc. 2009;57(5):553–6. 10.3200/JACH.57.5.553-556. 10.3200/JACH.57.5.553-556 19254898

[13] Karuniawati H, Putra ON, Wikantyasning ER. Impact of pharmacist counseling and leaflet on the adherence of pulmonary tuberculosis patients in lungs hospital in Indonesia. Indian J Tuberculosis [Internet]. 2019;66(3):364–9. 10.1016/j.ijtb.2019.02.015. 10.1016/j.ijtb.2019.02.015 31439181

[14] Yasin NM, Wahyono D, Riyanto BS, Sari IP, Farmasi F, Mada UG. Peningkatan peran apoteker dan outcome pasien tuberkulosis melalui uji coba model Training-Education-Monitoring-Adherence-Networking (TEMAN) apoteker: enhancing pharmacist’s role and tuberculosis patient outcomes through training-education-monitoring-adherence-networking. J Farmasi. 2017;6(4):247–66. 10.15416/ijcp.2017.6.4.247.

[15] Pradipta IS, Khairunnisa K, Bahar MA, Kausar MN, Fitriana E, Ruslami R, et al. Characteristics, knowledge, attitude, and practice of pharmacy personnel in supporting tuberculosis treatment: A multicenter cross-sectional study in a high-burden tuberculosis country. J Am Pharmacists Association [Internet]. 2024;64(3):102077. 10.1016/j.japh.2024.102077. 10.1016/j.japh.2024.102077 38537778

[16] Pradipta IS, Khairunnisa K, Bahar MA, Kausar MN, Fitriana E, Ruslami R, et al. Knowledge, attitude, and practice of community pharmacy personnel in tuberculosis patient detection: a multicentre cross-sectional study in a high-burden tuberculosis setting. BMJ Open. 2022;12(7):1–9. 10.1136/bmjopen-2021-060078. 10.1136/bmjopen-2021-060078 PMC9258488 35790331

[17] WHO & FIP. The role of pharmacists in tuberculosis care and control. WHO FIP joint statement. 2011. https://www.who.int/news/item/05-09-2011-signing-of-a-new-tuberculosis-initiative-between-the-world-health-organization-and-the-international-pharmaceutical-federation. Accessed 12 June 2025.

[18] Mardhiyyah CA, Aprilio K, Sumarheni, Gnanasan S, Pitaloka DAE, Pradipta IS. Can we involve pharmacists as direct service providers for people with tuberculosis? A narrative review of current evidence. Exploratory Res Clin Social Pharm. 2025;19(May):100613. 10.1016/j.rcsop.2025.100613. 10.1016/j.rcsop.2025.100613 PMC12152664 40502458

[19] Kemenkes RI. Panduan Penerapan Jejaring Layanan Tuberkulosis Di Fasilitas Kesehatan Pemerintah Dan Swasta Berbasis Kabupaten/Kota. District-Based Public-Private Mix/DPPM. Kementerian Kesehatan Republik Indonesia. 2019. https://www.studocu.id/id/document/universitas-malahayati/medical-student/ppm-1-navshs-jsvajkagn-nzjgaja/54421657. Accessed 10 May 2025.

[20] Kemenkes RI. Laporan Hasil Studi Inventori Tuberkulosis Indonesia 2023–2024. Kementerian Kesehatan Republik Indonesia; 2024.

[21] Kemenkes RI. Peraturan Menteri Kesehatan Republik Indonesia Nomor 19 Tahun 2024 Tentang Penyelenggaraan Pusat Kesehatan Masyarakat. Kementerian Kesehatan RI. 2024. https://peraturan.bpk.go.id/Details/312837/permenkes-no-19-tahun-2024. Accessed 12 February 2025.

[22] Kemenkes RI. Laporan Program Penanggulangan Tuberkulosis Tahun 2023. 2024. Available from: https://www.tbindonesia.or.id/pustaka---program-la/laporan-program-penanggulangan-tbc-2023/. Accessed 27 April 2025.

[23] Robert Wood Johnson Foundation. Lincoln and Guba’s evaluative criteria. Available from: http://www.qualres.org/HomeLinc-3684.html. Accessed 20 June 2025.

[24] Hennink M, Hutter I, Bailey A. Qualitative research methods. British library cataloguing in publication data. 2019.

[25] Flottorp SA, Oxman AD, Krause J, Musila NR, Wensing M, Godycki-Cwirko M, et al. A checklist for identifying determinants of practice: a systematic review and synthesis of frameworks and taxonomies of factors that prevent or enable improvements in healthcare professional practice. Implement Sci. 2013;8:35. 10.1186/1748-5908-8-35. 10.1186/1748-5908-8-35 PMC3617095 23522377

[26] Tong A, Sainsbury P, Craig J. Consolidated criteria for reporting qualitative research (COREQ): A 32-item checklist for interviews and focus groups. Int J Qual Health Care. 2007;19(6):349–57. 10.1093/intqhc/mzm042. 17872937 10.1093/intqhc/mzm042

[27] Kemenkes RI. Modul Pelatihan Jarak Jauh Manajemen Program Penanggulangan Tuberkulosis bagi Apoteker di Fasilitas Pelayanan Kesehatan. Kementerian Kesehatan Republik Indonesia. 2024. http://i-lib.ugm.ac.id/jurnal/download.php?dataId=8600.

[28] WHO. Guide to develop a national action plan on public-private mix for tuberculosis prevention and care. Geneva: WHO; 2017. p. 1–29. Available from: http://apps.who.int/bookorders.

[29] Nurfadila FS, Anggi JF, Rahmani SL, Pradipta IS, Destiani DP, Klinis F, et al. Barriers and strategies for latent tuberculosis treatment: a narrative review. J Farmako Bahari. 2025:204–33. 10.52434/jifb.v16i2.42065.

[30] Depkes RI. Pharmaceutical care tuberculosis. Departemen Kesehatan Republik Indonesia; 2005. https://drive.google.com/file/d/16FZ0voOkdgqiDiBcbycOv6J837hSg_G1/preview. Accessed 12 February 2025.

[31] Kemenkes RI. Petunjuk Teknis Standar Pelayanan Kefarmasian Di Apotik. Jakarta: Kementerian Kesehatan Republik Indonesia; 2019. https://repository.kemkes.go.id/book/557. Accessed 12 February 2025.

[32] Mardhiyyah CA, Zuniarto AA, Amanatin AD, Ryansyah H, Antonia V. Correlation of Community Pharmacist Knowledge, Attitudes, and Practices in the Detection of Tuberculosis Cases in West Java Province. Med Sains: Jurnal Ilmiah Kefarmasian. 2024;9(3):791–802. 10.37874/ms.v9i3.1296.

[33] Mardhiyyah CA, Zuniarto AA, Ryansyah H, Amanatin AD, Antonia V, Sumari S. Knowledge, Attitude, and Practice of Community Pharmacists in Monitoring Tuberculosis Patients from Provinces with High Prevalence of Tuberculosis in Indonesia. Jurnal Ilmiah Farmako Bahari. 2025;16(2):153–67. 10.52434/jifb.v16i2.42294.

[34] Ganachari MS, Rangaswamy UK, Knowledge. Attitude, Practice, and Implementation of community pharmacists’ role in treating tuberculosis patients in South India Region. Access Microbiol. 2020;2(2):162. 10.1099/acmi.fis2019.po0161.

[35] Minnery M, Contreras C, Pérez R, Solórzano N, Tintaya K, Jimenez J, et al. A cross-sectional study of knowledge and attitudes towards tuberculosis amongst front-line tuberculosis personnel in high burden areas of Lima, Peru. PLoS ONE. 2013;8(9):e74250. 10.1371/journal.pone.0075698. 10.1371/journal.pone.0075698 PMC3777965 24069437

[36] Abdulah R, Barliana MI, Pradipta IS, Halimah E, Diantini A, Lestari K. Assessment of patient care indicators at community pharmacies in Bandung City, Indonesia. Southeast Asian J Trop Med Public Health. 2014;45(5):1196–201. 25417523

[37] Kepmenkes RI. Keputusan Menteri Kesehatan Republik Indonesia Nomor 364/Menkes/Sk/V/2009 tentang Pedoman Nasional Penanggulangan Tuberkulosis (TB). 2009. https://repository.kemkes.go.id/book/124. Accessed 12 June 2025.

[38] PDPI. Tuberkulosis. Pedoman Diagnosis dan Penatalaksanaan di Indonesia Volume 1. Perhimpunan Dokter Paru Indonesia; 2021.

[39] Rahmadani I, Surjoputro A, Widjanarko B. Public-Private Mix Pada Program Pengendalian Tuberkulosis. Jurnal Kesmas (Kesehatan Masyarakat) Khatulistiwa. 2020;7(3):89. 10.29406/jkmk.v7i3.2080.

[40] Oliver SJ, Rattle A. Tuberculosis care in a low-incidence healthcare setting: what is the role of clinical pharmacists? J Pharm Pract Res. 2025;55(5):397–405. 10.1002/jppr.70009.

[41] Carter KL, Gabrellas AD, Shah S, Garland JM. Improved latent tuberculosis therapy completion rates in refugee patients through use of a clinical pharmacist. Int J Tuberculosis Lung Disease. 2017;21(4):432–7. 10.5588/ijtld.16.0575. 10.5588/ijtld.16.0575 28284259

[42] Syabariyah S, Pratiwi DE. The Family Role of Patient with Pulmonary TB About Prevention of Household Contacts Transmission in the Work Area of Puskesmas Perumnas II Pontianak. Atlantis Press. 2020;27:155–8. 10.2991/ahsr.k.200723.039.

[43] Parwitha IAA, Djunaidy VD, Alfian SD, Setyowibowo H, Pradipta IS. Psychosocial interventions to improve tuberculosis preventive treatment uptake and psychosocial outcomes: a systematic review. NPJ Prim Care Respir Med. 2025;35(1):40. 10.1038/s41533-025-00449-3. 41022732 10.1038/s41533-025-00449-3 PMC12480859

[44] Cahyani F, Dewi A. Stigma among tuberculosis patients: a bibliometric analysis and scoping review. Russian Open Med J. 2025;14(2). 10.15275/rusomj.2025.0209.

[45] Wang N, Wu L, Liu Z, Liu J, Liu X, Feng Y, et al. Influence of tuberculosis knowledge on acceptance of preventive treatment and the moderating role of tuberculosis stigma among China’s general population: cross-sectional analysis. BMC Public Health. 2024;24(1):2300. 10.1186/s12889-024-19812-z. 39180047 10.1186/s12889-024-19812-z PMC11344443

[46] Zhang H, Xin H, Du Y, Cao X, Pan S, Liu J, et al. Tuberculosis preventive treatment among individuals with inactive tuberculosis suggested by untreated radiographic abnormalities: a community-based randomized controlled trial. Emerg Microbes Infect. 2023;12(1):e2169195. 10.1080/22221751.2023.2169195. 36637403 10.1080/22221751.2023.2169195 PMC9888474

[47] Petri L. Concept analysis of interdisciplinary collaboration. Nurs Forum (Auckl). 2010;45(2):73–82. 10.1111/j.1744-6198.2010.00167.x. 10.1111/j.1744-6198.2010.00167.x 20536755

[48] Mustoor A, Alotaibi M, Abdullah I, Alsannat I, Alsewar TS, Alhaeerth YA. Interdisciplinary collaboration between nursing, emergency medicine, and pharmacy to improve patient outcomes. IJHS. 2018;2:539–54. 10.53730/ijhs.v2ns1.15454.

[49] Kurniasih DAA, Setiawati EP, Pradipta IS, Subarnas A. Interprofessional collaboration in the breast cancer unit: how do healthcare workers see it? BMC Womens Health. 2022;22(1):227. 10.1186/s12905-022-01818-7. 35698115 10.1186/s12905-022-01818-7 PMC9195208

[50] Tanvejsilp P, Loeb M, Dushoff J, Xie F. Healthcare Resource Uses and Out-of-Pocket Expenses Associated with Pulmonary TB Treatment in Thailand. Pharmacoecon Open. 2018;2(3):297–308. 10.1007/s41669-017-0053-0. 29623626 10.1007/s41669-017-0053-0 PMC6103920

[51] Scott NA, Sadowski C, Vernon A, Arevalo B, Beer K, Borisov A, et al. Using a medication event monitoring system to evaluate self-report and pill count for determining treatment completion with self-administered, once-weekly isoniazid and rifapentine. Cochrane database Syst reviews. 2023;129:107173. 10.1002/14651858.cd008451.pub2. 10.1016/j.cct.2023.107173 PMC11078335 37004811

[52] Holland DP, Sanders GD, Hamilton CD, Stout JE. Costs and cost-effectiveness of four treatment regimens for latent tuberculosis infection. Am J Respir Crit Care Med. 2009;179(11):1055–60. 10.1164/rccm.200901-0153oc. 19299495 10.1164/rccm.200901-0153OC PMC2689913

[53] do Prado TN, Wada N, Guidoni LM, Golub JE, Dietze R, Maciel ELN. Cost-effectiveness of community health worker versus home-based guardians for directly observed treatment of tuberculosis in Vitória, Espírito Santo State, Brazil. Cad Saude Publica. 2011;27(5):944–52. 10.1590/S0102-311X2011000500012. 21655845 10.1590/s0102-311x2011000500012 PMC3713773

[54] IAI. Panduan program apoteker bertamu. Ikatan apoteker Indonesia. 2024. https://id.scribd.com/document/773121666/Panduan-Apoteker-Bertamu. Accessed 10 May 2025.

[55] Thompson RR, Kityamuwesi A, Kuan A, Oyuku D, Tucker A, Ferguson O, et al. Cost and Cost-Effectiveness of a Digital Adherence Technology for Tuberculosis Treatment Support in Uganda. Value Health. 2022;25(6):924–30. 10.1016/j.jval.2021.12.002. 35667781 10.1016/j.jval.2021.12.002

[56] Union E. European Union. 2015. Sustainable Development Goals (SDGs). Available from: https://sdg2030indonesia.org/. Accessed 10 February 2025.

