# Supplementary material for: Integrating community pharmacists in tuberculosis infection care: challenges and strategic approaches in Indonesia
Source: BMC Health Serv Res. 2026 Feb 26;26:451. doi: 10.1186/s12913-026-14254-2 (PMC13041393; doi:10.1186/s12913-026-14254-2)
Supplement: Supplementary file 1 — Supplementary Material 1 [file 12913_2026_14254_MOESM1_ESM.pdf]

Supplementary File 1. Guidelines for an Interview

There are several steps for conducting an in-depth interview:

A. Introduction

Giving appreciation for participation in following this research

"Thank you, Sir/Madam, for taking the time to participate in the interview for this research."

B. Self-introduction

“Let me introduce myself, Cut Ainul Mardhiyyah, a doctoral student in the Pharmacy Doctoral program in the field of Pharmacology and Clinical Pharmacy. You can call me Cut Ainul. I will guide this interview by asking several questions related to the research.”

C. Identify the name, age, and background of the participants

"Before that, may I ask whether it is true that you are Mr./Mrs. (name)?"

“Yes, sir/madam, may I help you with your age and current profession?”

"Thank you, sir/madam, for the information."

D. Purpose of the interview

“Okay, Sir/Madam, before the interview, I would like to convey that the main objective of this study is to implement and evaluate systematically and structure the community pharmacist program for assisting in treating Tuberculosis (TB) patients in Indonesia. This study contains several specific objectives, namely:

1. *Build a strong coalition among stakeholders, namely professional organizations, such as IAI, city TB program managers, and academics, for the implementation of the Latent TB or the newest term TB infection (TBI) patient treatment assistance program.*
2. *Developing a community pharmacist model in pharmacies for TB preventive Therapy (TPT) assistance based on the local context.*
3. *Socialize the program to key actors, implementers, and stakeholders and increase pharmacists' capacity to assist in the treatment of individuals with TBI.*
4. *Implementing and evaluating a pilot study program for pharmacy pharmacists to assist in treating individuals with TBI.”*

E. Declaring that all interviews will be managed confidentially and used only for scientific purposes to improve TB treatment assistance in Indonesia.

“The conversations in this interview will be kept confidential and used only for scientific purposes to improve TB treatment assistance in Indonesia.”

F. States that the average duration of an interview will be around 60-120 minutes

“This interview will last for the next 60-120 minutes, depending on the topic and the various opinions that arise in the interview/discussion.”

G. Information about how the interview will be conducted, including a statement emphasizing that participants do not have to talk about anything they do not want to share and that participants can end the interview at any time.

“After you agree to participate in this study, we will include you as a participant. We will explore your ideas and opinions about implementing the community pharmacist program to assist in treating Tuberculosis (TB) patients in Indonesia. This exploration will be in the form of an in-depth interview.

In the preceding section, we first introduced the collaborative model designed for this study. The model begins with individuals diagnosed with TBI who receive TPT at the CHC and undergo an assessment to determine whether they require treatment support. For those who need assistance, patients may select a designated pharmacy where a community pharmacist will provide treatment support. Afterwards, the patient's TPT care shifts from the CHC to the chosen pharmacies.

Patients may collect their TPT medication every two weeks from the pharmacy or choose to visit weekly for medication pickup or direct dispensing. Individuals living with HIV who initiate TPT may receive online supervision or arrange alternative monitoring methods with the pharmacist before treatment initiation.

Community pharmacists are responsible for overseeing treatment progress, supporting medication adherence, monitoring adverse effects and potential drug interactions, providing TB education, screening for symptoms of active TB disease, and addressing potential drug-related problems (DRPs). These assessments are conducted throughout the three- to six-month treatment period, following guideline protocols, until the patient completes therapy. Throughout this process, CPs maintain regular communication with TB program staff and CHC pharmacists to ensure coordinated patient care, regarding patient treatment and care.

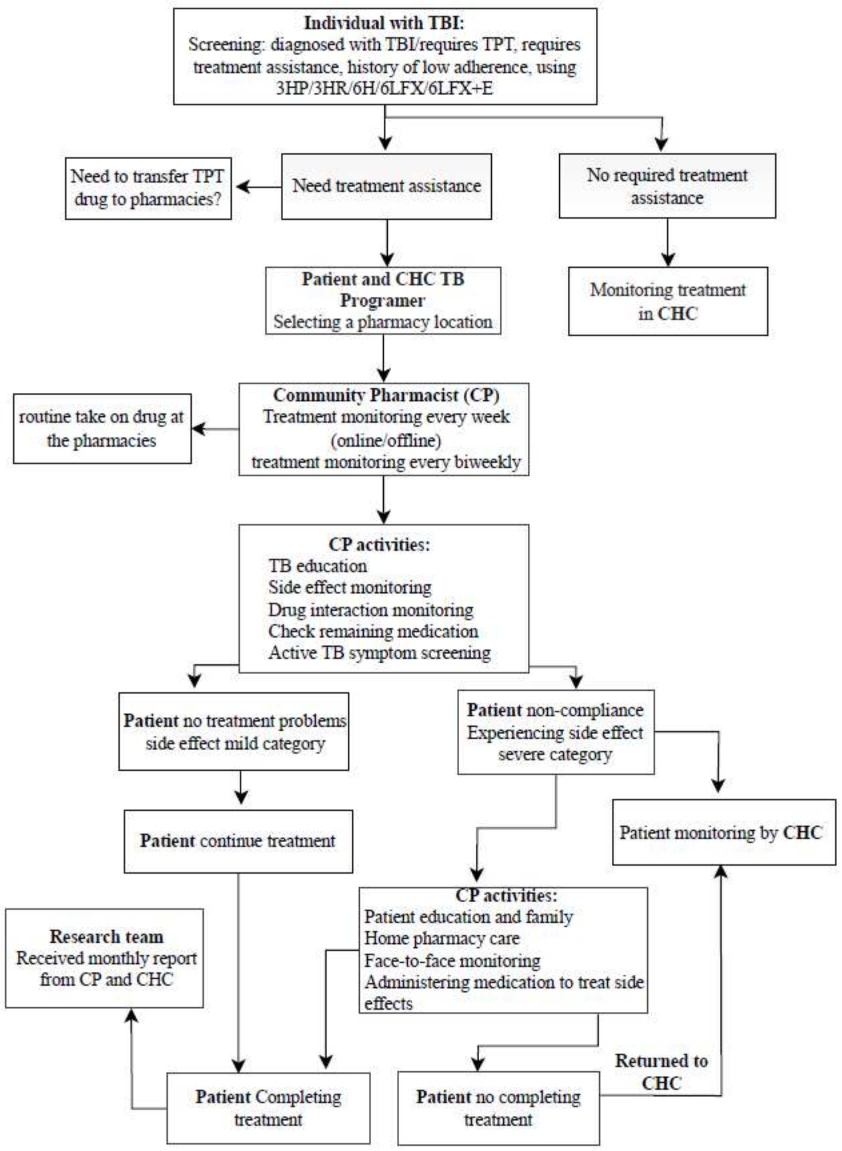

Once the program is developed with your input, it will be implemented at a pilot test site in the district. You will be involved as a supervisor in its implementation. As a supervisor, you will be invited once a month to a focus group discussion with the implementers (community pharmacists) to encourage and monitor them and provide your insights into the success of the program implementation.

Your participation in this study is voluntary. You are free to stop participating if you feel uncomfortable participating in this study."

H. Ensuring that participants understand the study and the informed consent form

"If you understand, please sign this informed consent form to indicate your willingness to be involved in this study."

I. Allow participants to ask questions about the study and the informed consent form.

"If you have any questions about this study, please read this sheet, or if anything is unclear, please contact the contact person."

Signing of the informed consent form.

J. Open questions

Open-ended questions will be asked of participants. The questions will ask about the factual conditions before the opinion. Questions can start with the general questions below:

1. *What is the current situation and achievements regarding TPT in individuals with TBI that you know of?*
2. *In your opinion, can community pharmacists have the role and responsibilities of a pharmacist in assisting individuals with TBI?*
3. *Can community pharmacists be optimal in assisting with the treatment of individuals with TBI? What are the challenges of the program offered?*
4. *What should be prepared for the implementation of this model program?*
5. *What is an effective strategy for assisting individuals with TBI treatment involving community pharmacists?*

K. *Closing Questions*

1. Offers additional comments on issues and expectations for improving the success of the program model offered.
"Do you have any additional comments regarding the model of assistance for individuals with TBI treatment by pharmacists at these pharmacies?"

- Statement of thanks
"Thank you for your willingness to participate in this interview. Hopefully, your input can improve this study and eradicate TB in Indonesia. Good morning/afternoon".

There are several steps for conducting the focus group discussion (FGD) or group interview (GI)**:**

A. Preparation
1. Determine the location of the FGD/GI and the equipment needed (e.g. recorder, camera, writing materials, mini-board, circle chairs)
2. Ensure participant criteria and the number of participants

B. Introduction
1. Introducing the moderators and assistant moderators
2. *"Let me introduce myself, Cut Ainul Mardhiyyah, a student of the Doctoral program in Pharmacology and Clinical Pharmacy. You can call me Cut Ainul. I will serve as the moderator of this focus group discussion by posing several questions related to the research. And at this time, I am also accompanied by my colleague as the assistant moderator, Firda."*
3. Explaining the background of the study and the purpose of the FGD/GI
"There are two main objectives of this FGD/GI, namely.
    1. *To develop a conceptual model of TBI treatment assistance by community pharmacists based on local context."*
    2. *To identify potential barriers, facilities, and strategies in the program of assisting the treatment of TBI patients by community pharmacists."*
4. Explaining the FGD/GI guidelines:

"I would like to explain a little about the provisions during this FGD/GI."

- There is no correct answer

  "It should be noted that in this FGD/GI, there are no wrong or right answers; participants are expected to be able to convey what they want to convey without hesitation."
- FGD/GI will be recorded

  "This interview will be recorded from start to finish for documentation purposes and will not be used for anything other than study purposes."
- Participants do not need to agree with other participants but should listen respectfully to other people's views.

  "In this discussion, participants do not need to agree with other participants but must respect and listen to the views of other participants."
- Declaring that all interviews will be managed confidentially and used only for scientific purposes to improve the care of TBI treatment in Indonesia.

  "The conversations in this interview will be managed confidentially and used only for scientific purposes to improve the care of TBI treatment in Indonesia."
- States that the average duration of an interview will be approximately 120 minutes.

  "This interview will last for the next 120 minutes, depending on the topic and the various opinions that arise in the interview/discussion."
- Information about how the interview will be conducted, including a statement emphasizing that participants do not have to talk about anything they do not want to share and that participants can end the interview at any time.

  "After you agree to participate in this study, we will include you as a participant. We will explore your ideas and opinions about implementing a community pharmacy program to assist in treating tuberculosis (TB) patients in Indonesia. This exploration will be in the form of FGD/GI). The FGD/GI will be conducted with other participants from diverse backgrounds (such as TB program implementers from health centers, community pharmacists, professional organizations, and TB Supervisors from the Health Office) to define a customized program based on the local context. Once the program is developed with your input, it will be implemented at a pilot site located in Cirebon City. You will be involved as a supervisor for the implementation of the program. As a supervisor, you will be invited once a month, together with the implementers, in a focus group discussion to encourage and monitor them and provide your insights for the success of the program implementation. The duration of the interview or discussion will be around 60-180 minutes. This depends on the topic and the various opinions that arise in the interview/discussion. Your participation in this study is voluntary. You are free to stop participating if you feel uncomfortable participating in this study."
- Ensure that participants understand the study and the informed consent form.

  "If you understand, I ask that you sign this informed consent form to indicate your willingness to be involved in this study."
- Allow participants to ask questions about the study and the informed consent form.

  "If you have any questions about this study, please read this sheet, or if anything is unclear in the future, please contact the contact person."
- Signing of informed consent form.

C. Introductory Questions

1. *After I explained the program earlier, in your opinion, is this model's program possible to implement?*
2. *Has the treatment of TBI achieved its treatment targets so far?*
3. *In your opinion, what is the optimal role of a pharmacist in assisting with TBI treatment?*
4. *What do you think about the responsibility of a pharmacist for assisting TPT?*
5. *Can pharmacists be optimal in assisting of individual with TBI treatment? How about the challenges in this program?*
6. *Is it necessary to prepare to have a TBI treatment assistance program for community pharmacists in the local and national context?*

D. Conclusion

In the closing session, the moderator will explain the conclusions for each objective and allow all participants to make their closing statements. The meeting will conclude with a "thank you" statement for the time and contributions of all participants.
