# Supplementary material for: Integrating community pharmacists in tuberculosis infection care: challenges and strategic approaches in Indonesia
Source: BMC Health Serv Res. 2026 Feb 26;26:451. doi: 10.1186/s12913-026-14254-2 (PMC13041393; doi:10.1186/s12913-026-14254-2)
Supplement: Supplementary file 2 — Supplementary Material 2 [file 12913_2026_14254_MOESM2_ESM.pdf]

Supplementary File 2. The coding process

Tema	Sub-tema	Kode	Kuotasi
GUIDELINE FACTOR	Supporting evidence	Lack of strength research evidence	*"There are no studies and guidelines for community pharmacists to assist patient TBI at this time."* Female, TB Programmer CHC.
		Practice guidance not yet adapted	*"Since each pharmacy and possibly CHC has an internal policy in practice, the guidelines and modules must be adapted to the local context in each pharmacy and CHC."* Female, pharmacist professional organization.
		Not regulated in National Guidelines	*"The program offered has not been regulated in the National TB guidelines, especially related to the mechanism of transferring drugs to pharmacies, which requires a policy from the Ministry of Health."* Female, District Health TB Supervisor.
INDIVIDUAL PHARMACIST	Knowledge and self-efficacy	Lack of knowledge about TB	*"For example, if we had prior knowledge about TB, we would feel confident." Because we lack knowledge, so yes, we are also not confident to handle it."* Female, CP.
		Lack of self-efficacy	
		Insufficient clinical training	*"We have not had any special training to handling ILTB patients, and We have never heard of heard that there is a term, Individual with TBI."*Female, CP.
	Adaptability to the local context	Programs disrupt workflow	"*I am the only pharmacist in the pharmacies, and I have other responsibilities outside of this job*." Female, CP.
PATIENT FACTOR	Need for assistance	Variable patient need	*"In terms of assistance, I have observed that one patient appreciates receiving a daily message on WhatsApp, while another patient prefers not to receive such messages."* Female, TB Programmer.
	Patient beliefs and understanding	Limited understanding of CPs' roles	*"Yes, when I go to the pharmacies, I find him, not the same pharmacist, so I don't know him."* Men, individuals with ILTB.
		Additional services accessed	*"The program offered has not been regulated in the National TB guidelines, especially related to the mechanism of transferring drugs to pharmacies, which requires a policy from the Ministry of Health*." Female, District Health TB Supervisor.
PROFESSIONAL INTERACTIONS	Team communication	Poor communication for interdisciplinary management TBI	*"The pharmacies have not been exposed by any CHC program so far."* Female, CP.
		Weak integration CPs in TPT program	*"Because there is no regulation, and it is not yet possible to integrate CP in TBI treatment, it is also worrying that they have not been able to manage patients properly, especially patients who are lost to follow-up."* Female, District Health TB Supervisor.
	Refferal	Difficulty referring patients	"*CHC will find it hard to refer patients to get the right education and reasons to be referred due to the constraints of patients who do not want to visit two places."* Female, District Health TB Supervisor.
INCENTIVES AND RESOURCES	Incentives for pharmacists	No funding for CP's assistance	*"Well, the funded transportation is only active patient assistance by cadres."* Female, CHC TB Programmer.
	Human resources	Staffing shortages	*"I am the only pharmacist in the pharmacies, because it is also a small pharmacy, so I take care of everything myself*." Female, CP.
	Information	Pharmacies no access	*"Access to SITB is limited to CHC and hospitals. This program*

	systems	to SITB for reporting	*will be constrained by recording reporting through SITB because the patient referral system can only be carried out at health facilities registered with SITB. As of now, there is no rule regarding whether pharmacies outside of the CHC can access SITB."* Female, District Health TB Programmer.
CAPACITY FOR ORGANIZATIONAL CHANGE	Regulations, organizational policies	Lack of organizational support structur	*"Because there has been no circular or warning from professional organizations that the pharmacies must be involved."* Female, Professional Organization of Pharmacists.
	Priority of necessary changes	CP involvement has not been a priority	"*Yes, because we also don't know what programs are at the CHC, including the TB program."* Female, CP.
SOCIAL, POLITICAL, AND LEGAL FACTOR	Legislation	Regulatory and policy limitation	*"There is no policy allowing CPs to assist in the treatment of TBI patients; however, assistance is available for cadres, NGOs, and other health workers."* Female, District Health TB Programmer.
	Logistics	Unstable logistic	"*The logistics—that's the obstacle, that's why I don't dare to promote TPT anymore; if the logistics don't exist, we are ashamed of the patient*." Female, CHC TB Programmer.
		Drug transfer mechanism	"*Yes, will CP take the TPT drug from CHC? Alternatively, the patient might bring the TPT drug from the CHC to the pharmacies, which is also confusing. Don't let the patient come here later. The medicine is not in the pharmacies*." Female, Pharmacist Professional Organization.
		Medication's side effects	*"If the patient feels side effects that require medication, can the medicine be free at the pharmacies? Therefore, we recommend that the CHC distribute the medication to the pharmacies to manage TPT side effects*." Female, Pharmacist Professional Organization.
