# Supplementary material for: Integrating community pharmacists in tuberculosis infection care: challenges and strategic approaches in Indonesia
Source: BMC Health Serv Res. 2026 Feb 26;26:451. doi: 10.1186/s12913-026-14254-2 (PMC13041393; doi:10.1186/s12913-026-14254-2)
Supplement: Supplementary file 3 — Supplementary Material 3 [file 12913_2026_14254_MOESM3_ESM.pdf]

Supplementary File 3. Code's pattern among the study participants

No.	Codes	Supervisor TB in District	CHC TB Programmer	Pharmacist in CHC	Community Pharmacist	Head of CHC	Doctor in CHC	Organiza-tional Pharmacist	Patient with ILTB	Total
CHALLENGES										
1	GUIDELINE FACTOR									
	Lack of strength research evidence	1			1			2		4
	Practice guidance not yet adapted				2			1		3
	Not regulated in National Guidelines	1				2	1			4
2	INDIVIDUAL PHARMACIST									
	Lack of knowledge about TB				2			1		3
	Lack of self-efficacy			1	2					3
	Insufficient Clinical Training			1	1			1		3
	Program disrupt workflow			1	2			2		5
3	PATIENT FACTOR									
	Variable patient needs		2	1	1				2	6
	Limited understanding of CPs' roles				1				2	3
	Additional services accessed	2	1							3
4	PROFESSIONAL INTERACTIONS									
	Poor communication for interdisciplinary management TBI	1	5	4	2			2		14
	Difficulty referring patients	1								1
	Weak integration of CPs in TPT program	2	1	2	1	3	3			12
5	INCENTIVES AND RESOURCES									
	No funding for CPs' assistance	2	2	3	2			2		11
	Staffing shortages				2			1		3
	No access to SITB for reporting	2	2							4
6	CAPACITY FOR ORGANIZATIONAL CHANGE									
	Lack of Organizational support structure			2	2			1		5

	CP involvement has not been a priority			1	2			1		4
7	SOCIAL, POLITICAL, AND LEGAL FACTORS									
	Regulatory and policy limitations	1			1	1				3
	Unstable logistic		1	1						2
	Drug transfer mechanism	2	2		1		1			6
	Medication’s side effect		1	1	1			2		5
STRATEGIES										
1	Clearly create guidelines for evaluation	1	1					1		3
2	Develop effective collaboration systems		2			1		1		4
3	Establish an effective patient counselling and education program						1		2	3
4	Cross-sector support		2	1		1			1	5
5	Explore alternative funding				1	1		1		3
6	Program socialization		2		2	1	1			6
7	Ensure that TBI drugs and tests are readily available		3	2		3				8
TOTAL										139
